# *Hypoxylon luteogranulatum* (Hypoxylaceae, Xylariales), a novel species from Thailand with distinct chemical and ecological traits

**DOI:** 10.1080/21501203.2024.2435979

**Published:** 2025-01-16

**Authors:** Sarunyou Wongkanoun, Esteban Charria-Girón, Marjorie Cedeño-Sanchez, Boonchuai Chainuwong, Sayanh Somrithipol, Eric Kuhnert, Prasert Srikitikulchai, Natapol Pornputtapong, Frank Surup, Jennifer Luangsa-ard, Marc Stadler

**Affiliations:** a Department of Biochemistry and Microbiology, Center of Excellence for DNA Barcoding of Thai Medicinal Plants, Faculty of Pharmaceutical Sciences, Chulalongkorn University, Bangkok, Thailand; b National Biobank of Thailand (NBT), National Center for Genetic Engineering and Biotechnology (BIOTEC), Pathum Thani, Thailand; c Department of Microbial Drugs, Helmholtz Centre for Infection Research GmbH (HZI), German Centre for Infection Research Association (DZIF), Braunschweig, Germany; d Institute of Microbiology, Technische Universität Braunschweig, Braunschweig, Germany; e Plant Microbe Interaction Research Team (APMT), National Center for Genetic Engineering and Biotechnology (BIOTEC), Pathum Thani, Thailand; f Ginkgo Bioworks, Inc., Boston, USA

**Keywords:** Phylogeny, tetramic acids, metabolomics, genome mining, Sordariomycetes: new species, Xylariales

## Abstract

During the course of our ongoing study of the diversity of Thai fungi and their secondary metabolites, numerous specimens within the Hypoxylaceae have been characterised by traditional morphology, chemotaxonomy using ultra-high-performance liquid chromatography coupled to diode array detection and ion mobility tandem mass spectrometry (UHPLC-DAD-IM-MS/MS), and molecular phylogenetic analyses. MS/MS-based analysis of the major stromatal metabolites of a newly identified taxon, *Hypoxylon luteogranulatum*, indicated the production of distinct compounds compared to the azaphilone and binaphthalene pigments commonly found in the Hypoxylaceae, aside from the presence of the chemotaxonomic marker binaphthalene tetrol (BNT). Further analysis suggested that one of the major metabolites had the molecular formula C_13_H_13_NO_3_, identical to hypoxyvermelhotin A, a yellow pigment so far exclusively found in *Hypoxylon lechatii*. Its identity was confirmed after purification by preparative high-performance liquid chromatography with nuclear magnetic resonance (NMR) data, and genome analysis of *H. lechatii* revealed the presence of different hybrid polyketide synthases-non-ribosomal peptide synthetases (PKS-NRPS) hybrid clusters. Despite chemotaxonomic similarities with *H. lechatii*, we propose a new species, which is morphologically distinct from *H. lechatii*. Our molecular phylogenetic study provides substantial evidence distinguishing *H. luteogranulatum* clearly from *H. lechatii* and allied members within the Hypoxylaceae. Additionally, future studies are needed to better understand the ecological behaviour of *H. luteogranulatum* and identify the ecological role of the vermelhotin-like molecules within this putative interaction.

## GRAPHICAL ABSTRACT

**Figure:**
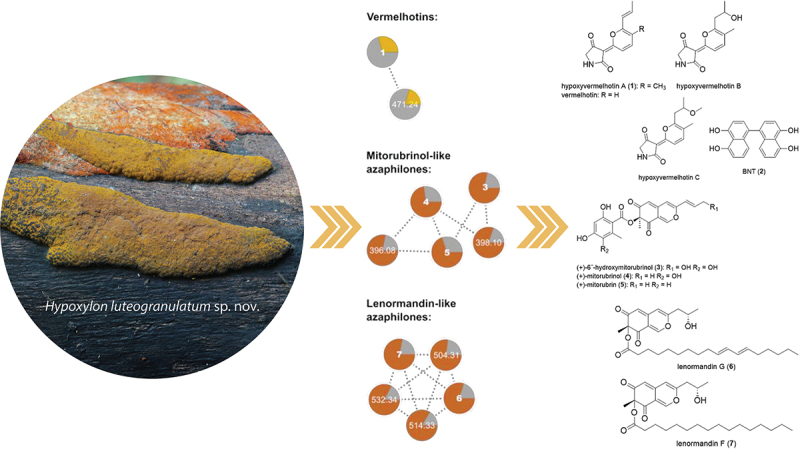

## 1. Introduction

The genus *Hypoxylon* is one of the largest in the Hypoxylaceae. Ju and Rogers (1996) discovered that the phenotypic features including asexual morph characters, stromatal KOH-extractable pigments, as well as the ascospores and asci constitute valuable diagnostic characters. Most of the Xylariales, including all hypoxyloid taxa, have been traditionally regarded as saprobes on wood but also include species frequently reported as endophytes and even from coprophilous sources (Stadler et al. 2014). Several species have eventually been identified on the basis of their asexual morph structures and their characteristic lifestyle (Bills et al. 2012; Pažoutová et al. 2013) or based on polyphasic taxonomy, including the comparisons of morphological and molecular data (Hsieh et al. 2005) as well as the comparison between traditional morphology with chemotaxonomic data (Fournier et al. 2010). Additionally, the stromatal secondary metabolite profiling generated by high-performance liquid chromatography coupled with diode array detection and mass spectrometry (HPLC-DAD/MS) not only proved highly useful for the discrimination of species but even led to the discovery of a plethora of novel natural products with prominent biological activities (Quang et al. 2004; Helaly et al. 2018).

Recently, the taxonomic relationships between *Hypoxylon* and related genera were confirmed by polyphasic taxonomic methodologies, which include the comparison of phenotypic, molecular, and chemotaxonomic data (Wendt et al. 2018). These studies examined different specimens and authentic strains of the Xylariales, resulting in a reorganisation of their genera and the establishment of a phylogenetic backbone for these Pyrenomycetes. In a multigene phylogenetic analysis of the Hypoxylaceae, *Hypoxylon* was divided into several clades. *Hypoxylon fragiforme*, the type species of *Hypoxylon*, was shown as a member of a small clade, which included *H. haematostroma* (Wendt et al. 2018). The major *Hypoxylon* clade is represented by species of *H. rubiginosum* sensu stricto (Ju and Rogers 1996). The secondary metabolites found in the stromata of these taxa, which mostly belong to the azaphilone or binaphthalene compound classes are useful for the discrimination at the genus or species level (Bitzer et al. 2008; Pourmoghaddam et al. 2020).

During our extensive fieldwork and taxonomic studies on Xylariales in Thailand, we discovered a new species in the region with a rare phylogenetic placement within the Hypoxylaceae. The present study aims to report and discuss the implications of the new species phylogenetic relationships based on multi-locus phylogenetic analysis, as well as its description and illustration. In addition, we investigated its stromatal secondary metabolites by state-of-the-art metabolomics and genomic analyses.

## 2. Materials and methods

### 2.1. Sample collection and cultivation

The studied specimens were collected in the community forest in northern Thailand. Photographs were taken in their natural habit using a 60D digital camera (Canon, Tokyo, Japan). The cultures were isolated using multiple spore isolation techniques as described in Sir et al. (2016). After a couple of days of incubation, the hyphal tips were transferred to new agar plates. Axenic cultures were deposited in the BIOTEC Culture Collection (BCC) and National Biobank of Thailand (NBT) and the dried voucher specimens were kept in the BIOTEC Bangkok Herbarium (BBH), Thailand, respectively.

### 2.2. Morphological characterisation, HPLC profiling, and compound isolation

Morphological characters, such as stromatal size and shapes, perithecia, asci, and ascospores, were examined following Stadler et al. (2014) using a compound microscope Olympus Z×31 (Olympus Corporation, Tokyo, Japan) and a stereo microscope Olympus SZ61 (Olympus Corporation, Tokyo, Japan). Fungal cultures were grown on several media *i.e*. Oatmeal Agar (Difco: OA), Potato Dextrose Agar (Difco: PDA). Morphological studies were carried out on 9 cm Petri dishes. Conidiogenous cells and conidiophore branching patterns of the asexual morph were investigated as proposed by Ju and Rogers (1996). Furthermore, stromatal colour, KOH-extractable pigments, and cultures are determined according to the colour chart of Rayner (1970) and the colour codes proposed in this chart are given. For chemotaxonomic studies, the stromatal secondary metabolites were extracted with acetone and analysed using UHPLC-DAD-IM-MS/MS in a similar manner as described by Cedeño-Sanchez et al. (2023, 2024b).

For the purification of the major secondary metabolites, the stromatal material was detached from its substrate and extracted three times with acetone in an ultrasonic batch for 30 min at 40 °C. The resulting solution was filtered and evaporated to yield 50 mg of stromatal extract. This was purified by preparative reversed-phase (RP)-HPLC (VP Nucleodur 100-10 C18 ec column (150 mm × 40 mm; Macherey-Nagel, Düren, Germany), solvent A: H_2_O +0.1% formic acid, solvent B: ACN + 0.1% formic acid, flow rate 40 mL/min and UV detection at 210, 240, and 300 nm, gradient: From 30% to 75% B in 50 min, from 75% to 100% in 15 min, and 100% B isocratic for 10 min) to afford hypoxyvermelhotin (**1**) (4.99 mg, tR = 19–21 min).

### 2.3. DNA extraction, polymerase chain reaction (PCR), and sequencing

A method based on cetyltrimethylammonium bromide (CTAB) was used to extract total genomic DNA from the mycelia according to Mongkolsamrit et al. (2009). The internal transcribed spacer regions (ITS) and large subunit of the rDNA (LSU), RNA polymerase II (*RPB2*), and beta-tubulin (*TUB2*) were amplified, following the standard primers introduced by White et al. (1990; ITS1, ITS4, and ITS5), Vilgalys and Hester (1990; LR5 and LROR), Liu et al. (1999; *RPB2*-5F and 7Cr) and O’Donnell and Cigelnik (1997; T1 and T22), according to the protocols of Wendt et al. (2018). PCR was conducted in 25 µL reaction volumes consisting of 1× PCR buffer, 200 μmol/L of each of the four dNTPs, 2.5 mmol/L MgCl_2_, 1 U *Taq* DNA Polymerase recombinant (Thermo Scientific, USA), 0.5 µmol/L of each primer, and 50–100 ng of DNA template. The PCR conditions were performed as follows: 94 °C for 2 min, followed by 35 cycles of denaturation at 94 °C for 1 min, annealing at a suitable temperature for 1 min, extension at 72 °C for 2 min, and a final extension of 72 °C for 10 min. The annealing temperature of each gene was 55 °C for ITS and LSU; 54 °C for *RPB2*; and 53 °C for *TUB2*. The PCR products were sent to Macrogen Co. (Seoul, Korea) for purification and sequencing using the same primers used for the PCR amplification reaction. DNA sequences were checked and assembled using BioEdit v. 7.2.5 (Hall 1999). All newly generated sequences were submitted to GenBank (https://www.ncbi.nlm.nih.gov/) and listed in Table 1.

**Table 1.** List of all taxa used in the current phylogenetic study.

Species		Strains	Origin	GenBank accession numbers				Reference	Status
				ITS	LSU	*RPB2*	*TUB2*		
*Annulohypoxylon annulatum*		CBS 140775	Texas	KY610418	KY610418	KY624263	KX376353	Kuhnert et al. (2017a; *TUB2*), Wendt et al. (2018; ITS, LSU, *RPB2*)	ET
*A. michelianum*		CBS 119993	Spain	KX376320	KY610423	KY624234	KX271239	Kuhnert et al. (2014a; ITS, *TUB2*), Wendt et al. (2018; LSU, *RPB2*)	
*A. truncatum*		CBS 140778	Texas	KY610419	KY610419	KY624277	KX376352	Kuhnert et al. (2017a; *TUB2*), Wendt et al. (2018; ITS, LSU, *RPB2*)	
*Daldinia bambusicola*		CBS 122872	Thailand	KY610385	KY610431	KY624241	AY951688	Hsieh et al. (2005; *TUB2*), Wendt et al. (2018; ITS, LSU, *RPB2*)	
*Dal. childiae*		CBS 122881	France	KU683757	MH874773	KU684290	KU684129	U’Ren et al. (2016; ITS, *TUB2, RPB2*), Vu et al. (2019; LSU)	
*Dal. concentrica*		CBS 113277	Germany	AY616683	KY610434	KY624243	KC977274	Triebel et al. (2005; ITS), Kuhnert et al. (2014a; *TUB2*), Wendt et al. (2018; LSU, *RPB2*)	ET
*Dal. dennisii*		CBS 114741	Australia	JX658477	KY610435	KY624244	KC977262	Stadler et al. (2014; ITS), Kuhnert et al. (2014a; *TUB2*), Wendt et al. (2018; LSU, *RPB2*)	HT
*Dal. eschscholtzii*		MUCL 45435	Benin	JX658484	KY610437	KY624246	KC977266	Stadler et al. (2014; ITS), Kuhnert et al. (2014a; *TUB2*), Wendt et al. (2018; LSU, *RPB2*)	HT
*Dal. petriniae*		MUCL 49214	Austria	AM749937	KY610439	KY624248	KC977261	Bitzer et al. (2008; ITS), Kuhnert et al. (2014a; *TUB2*), Wendt et al. (2018; LSU, *RPB2*)	
*Dal. placentiformis*		MUCL 47603	Mexico	AM749921	KY610440	KY624249	KY204016	Stadler et al. (2014; ITS), Kuhnert et al. (2014a; *TUB2*), Wendt et al. (2018; LSU, *RPB2*)	
*Dal. vernicosa*		CBS 119316	Germany	KY610395	KY610442	KY624252	KC977260	Kuhnert et al. (2014a; *TUB2*), Wendt et al. (2018; ITS, LSU, *RPB2)*	ET
*Durotheca crateriformis*		GMBC 0205	China	MH645426	MH645425	MH645427	MH049441	de Long et al. (2019)	T
*D. guizhouensis*		GMBC 0065	China	MH645423	MH645421	MH645422	MH049439	de Long et al. (2019)	T
*D. rogersii*		GMBC 0204	China	MH645433	MH645434	MH645435	MH049449	de Long et al. (2019)	
*D. rogersii*		YMJ 92031201	Taiwan of China	MH645433	MH645434	MH645435	MH049449	Ju et al. (2007)	ET
*Graphostroma platystomum*		CBS 270.87	France	JX658535	DQ836906	KY624296	HG934108	Zhang et al. (2006; LSU), Stadler et al. (2014; ITS), Koukol et al. (2015; *TUB2*), Wendt et al. (2018; *RPB2*)	T
*Hypomontagnella barbarensis*		STMA 14081	Argentina	MK131720	MK131718	MK135891	MK135893	Lambert et al. (2019)	T
*Hy. monticulosa*		MUCL 54604	French Guiana	KY610404	KY610487	KY624305	KX271273	Wendt et al. (2018)	ET
*Hy. submonticulosa*		CBS 115280	France	KC968923	KY610457	KY624226	KC977267	Kuhnert et al. (2014a; ITS, *TUB2*), Wendt et al. (2018; LSU, *RPB2*)	
*Hypoxylon addis*		MUCL 52797	Ethiopia	KC968931	ON954141	OP251037	KC977287	Kuhnert et al. (2014a; ITS, *TUB2*), Cedeño-Sanchez et al. (2023; LSU, *RPB2*)	T
*H. aveirense*		MUM 19.40	Portugal	MN053021	ON954142	OP251028	MN066636	Vicente et al. (2021; ITS, *TUB2*), Cedeño-Sanchez et al. (2023; LSU, *RPB2*)	
*H. baruense*		UCH9545	Panama	MN056428	ON954143	N/A	MK908142	Cedeño-Sanchez et al. (2020; ITS, *TUB2*), Cedeño-Sanchez et al. (2023; LSU, *RPB2*)	
*H. canariense*		MUCL 47224	Spain	ON792787	ON954140	OP251029	ON813073	Cedeño-Sanchez et al. (2023)	PT
*H. carneum*		MUCL 54177	France	KY610400	KY610480	KY624297	KX271270	Wendt et al. (2018)	
*H. cercidicola*		CBS 119009	France	KC968908	KY610444	KY624254	KC977263	Kuhnert et al. (2014a; ITS, *TUB2*), Wendt et al. (2018; LSU, *RPB2*)	
*H. chionostomum*		STMA 14060	Argentina	KU604563	ON954144	OP251030	ON813072	Sir et al. (2016; ITS); Cedeño-Sanchez et al. (2023; LSU, *RPB2*)	
*H. chrysalidosporum*		FCATAS2710	China	OL467294	OL615106	OL584222	OL584229	Ma et al. (2022)	T
*H. crocopeplum*		CBS 119004	France	KC968907	KY610445	KY624255	KC977268	Kuhnert et al. (2014a; ITS, *TUB2*), Wendt et al. (2018; LSU, *RPB2*)	
*H. cyclobalanopsidis*		FCATAS2714	China	OL467298	OL615108	OL584225	OL584232	Ma et al. (2022)	T
*H. erythrostroma*		MUCL 53759	Martinique	KC968910	ON954154	OP251031	KC977296	Kuhnert et al. (2014a; ITS2, *TUB*), Cedeño-Sanchez et al. (2023; LSU, *RPB2*)	
*H. eurasiaticum*		MUCL 57720	Iran	MW367851	N/A	MW373852	MW373861	Lambert et al. (2021)	T
*H. fendleri*		MUCL 54792	French Guiana	KF234421	KY610481	KY624298	KF300547	Kuhnert et al. (2014a; ITS, *TUB2*), Wendt et al. (2018; LSU, *RPB2*)	
*H. ferrugineum*		CBS 141259	Austria	KX090079	N/A	N/A	KX090080	Friebes and Wendelin (2016)	
*H. fragiforme*		MUCL 51264	Germany	KC477229	KM186295	MK887342	KX271282	Stadler et al. (2013; ITS), Daranagama et al. (2015; LSU, *RPB2*), Wendt et al. (2018; *TUB2*)	ET
*H. fuscoides*		MUCL 52670	France	ON792789	ON954145	OP251038	ON813076	Cedeño-Sanchez et al. (2023)	T
*H. fuscum*		CBS 113049	Germany	KY610401	KY610482	KY624299	KX271271	Wendt et al. (2018)	ET
*H. gibriacense*		MUCL 52698	France	KC968930	ON954146	OP251026	ON813074	Kuhnert et al. (2014a; ITS), Cedeño-Sanchez et al. (2023; LSU, *RPB2, TUB2*)	T
*H. griseobrunneum*		CBS 331.73	India	KY610402	KY610483	KY624300	KC977303	Kuhnert et al. (2014a; *TUB2*), Wendt et al. (2018; ITS, LSU, *RPB2*)	T
*H. guilanense*		MUCL 57726	Iran	MT214997	MT214992	MT212235	MT212239	Pourmoghaddam et al. (2020)	T
*H. haematostroma*		MUCL 53301	Martinique	KC968911	KY610484	KY624301	KC977291	Wendt et al. (2018; LSU, *RPB2*), Kuhnert et al. (2014a; ITS, *TUB2*)	ET
*H. hainanense*		FCATAS2712	China	OL467296	OL616132	OL584224	OL584231	Ma et al. (2022)	T
*H. hinnuleum*		ATCC 36255	USA	MK287537	MK287549	MK287562	MK287575	Sir et al. (2019)	T
*H. howeanum*		MUCL 47599	Germany	AM749928	KY610448	KY624258	KC977277	Bitzer et al. (2008; ITS), Kuhnert et al. (2014a; *TUB2*), Wendt et al. (2018; LSU, *RPB2*)	
*H. hypomiltum*		MUCL 51845	Guadeloupe	KY610403	KY610449	KY624302	KX271249	Wendt et al. (2018)	
*H. invadens*		MUCL 51475	France	MT809133	MT809132	MT813037	MT813038	Becker et al. (2020)	T
*H. investiens*	CBS 118183		Malaysia	KC968925	KY610450	KY624259	KC977270	Kuhnert et al. (2014a; ITS, *TUB2*), Wendt et al. (2018; LSU, *RPB2*)	
*H. isabellinum*	MUCL 53308		Martinique	KC968935	ON954155	OP251032	KC977295	Kuhnert et al. (2014a; ITS, *TUB2*), Cedeño-Sanchez et al. (2023; LSU, *RPB2*)	T
*H. laschii*	MUCL 52796		France	JX658525	ON954147	OP251027	ON813075	Stadler et al. (2014; ITS), Cedeño-Sanchez et al. (2023; LSU, *RPB2, TUB2*)	
*H. lateripigmentum*	MUCL 53304		Martinique	KC968933	KY610486	KY624304	KC977290	Kuhnert et al. (2014a; ITS, *TUB2*), Wendt et al. (2018; LSU, *RPB2*)	T
*H. lechatii*	MUCL 54609		French Guiana	KF923407	ON954148	OP251033	KF923405	Kuhnert et al. (2014b; ITS, *TUB2*), Cedeño-Sanchez et al. (2023; LSU, *RPB2*)	T
*H. lenormandii*	CBS 119003		Ecuador	KC968943	KY610452	KY624261	KC977273	Kuhnert et al. (2014a; ITS, *TUB2*), Wendt et al. (2018; LSU, *RPB2*)	
*H. lienhwacheense*	MFLUCC 14-1231		Thailand	KU604558	MK287550	MK287563	KU159522	Sir et al. (2016; ITS, *TUB2*), Sir et al. (2019; LSU, *RPB2*)	
*H. lividipigmentum*	STMA14045		Argentina	ON792788	ON954149	N/A	ON813077	Cedeño-Sanchez et al. (2023)	
*H. lividipigmentum*	BCRC 34077		Mexico	JN979433	N/A	N/A	AY951735	Hsieh et al. (2005)	IT
***H. luteogranulatum***	**BCC 79720**		**Thailand**	**PP955304**	**PP955701**	**PP968827**	**PQ000351**	**This study**	**HT**
***H. luteogranulatum***	**BCC 91230**		**Thailand**	**PP955305**	**PP955702**	**PP968829**	N/A	**This study**	
***H. luteogranulatum***	**BCC 91232**		**Thailand**	**PP955306**	**PP955703**	**PP968828**	N/A	**This study**	
*H. macrocarpum*	CBS119012		Germany	ON792785	ON954151	OP251034	ON813071	Cedeño-Sanchez et al. (2023)	
*H. munkii*	MUCL 53315		Martinique	KC968912	ON954153	OP251035	KC977294	Kuhnert et al. (2014a; ITS, *TUB2*), Cedeño-Sanchez et al. (2023; LSU, *RPB2*)	
*H. musceum*	MUCL 53765		Guadeloupe	KC968926	KY610488	KY624306	KC977280	Kuhnert et al. (2014a; ITS, *TUB2*), Wendt et al. (2018; LSU, *RPB2*)	
*H. ochraceum*	MUCL 54625		Martinique	KC968937	N/A	KY624271	KC977300	Kuhnert et al. (2014a; ITS, *TUB2*), Wendt et al. (2018; *RPB2*)	ET
*H. olivaceopigmentum*	DSM 107924		USA	MK287530	MK287542	MK287555	MK287568	Sir et al. (2019)	T
*H. perforatum*	CBS115281		France	KY610391	KY610455	KY624224	KX271250	Wendt et al. (2018)	
*H. petriniae*	CBS 114746		France	KY610405	KY610491	KY624279	KX271274	Wendt et al. (2018)	T
*H. pilgerianum*	STMA 13455		Martinique	KY610412	KY610412	KY624308	KY624315	Wendt et al. (2018)	
*H. porphyreum*	CBS 119022		France	KC968921	KY610456	KY624225	KC977264	Kuhnert et al. (2014a; ITS, *TUB2*), Wendt et al. (2018; LSU, *RPB2*)	
*H. pseudofuscum*	DSM112038		Germany	MW367857	MW367848	MW373858	MW373867	Lambert et al. (2021)	T
*H. pulicicidum*	CBS 122622		Martinique	JX183075	KY610492	KY624280	JX183072	Bills et al. (2012; ITS, *TUB2*), Wendt et al. (2018; LSU, *RPB2*)	T
*H. rickii*	MUCL 53309		Martinique	JX183075	KY610492	KY624280	JX183072	Kuhnert et al. (2014a; ITS, *TUB2*), Wendt et al. (2018; LSU, *RPB2*)	T
*H. rubiginosum*	MUCL 52887		Martinique	KC968932	KY610416	KY624281	KC977288	Stadler et al. (2013; ITS), Wendt et al. (2018; *TUB2*, LSU, *RPB2*)	ET
*H. samuelsii*	MUCL 51843		Germany	KC477232	KY610469	KY624266	KY624311	Kuhnert et al. (2014a; ITS, *TUB2*), Wendt et al. (2018; LSU, *RPB2*)	ET
*H. sporistriatatunicum*	UCH 9542		Guadeloupe	KC968916	KY610466	KY624269	KC977286	Cedeño-Sanchez et al. (2020; ITS, *TUB2*); Cedeño-Sanchez et al. (2023; LSU, *RPB2*)	ET
*H. subticinense*	MUCL 53752		French Guiana	KC968913	ON954152	N/A	KC977297	Kuhnert et al. (2014a; ITS, *TUB2*), Cedeño-Sanchez et al. (2023; LSU, *RPB2*)	
*H. texense*	DSM 107933		USA	MK287536	MK287548	MK287561	MK287574	Sir et al. (2019)	T
*Hypoxylon ticinense*	CBS 115271		France	JQ009317	KY610471	KY624272	AY951757	Hsieh et al. (2005; ITS, *TUB2*), Wendt et al. (2018; LSU, *RPB2*)	
*H. trugodes*	MUCL 54794		Sri Lanka	KF234422	KY610493	KY624282	KF300548	Kuhnert et al. (2014a; ITS, *TUB2*), Wendt et al. (2018; LSU, *RPB2*)	ET
*H. vogesiacum*	CBS 115273		France	KC968920	KY610417	KY624283	KX271275	Kuhnert et al. (2014a; ITS), Kuhnert et al. (2017a; *TUB2*), Wendt et al. (2018; LSU, *RPB2*)	
*H. wuzhishanense*	FCATAS2708		China	OL467292	OL615104	OL584220	OL584227	Ma et al. (2022)	T
*Jackrogersella cohaerens*	CBS 119126		Germany	KY610396	KY610497	KY624270	KY624314	Wendt et al. (2018)	
*J. multiformis*	CBS 119016		Germany	KC477234	KY610473	KY624290	KX271262	Kuhnert et al. (2014a; ITS), Kuhnert et al. (2017a; *TUB 2*), Wendt et al. (2018; LSU, *RPB2*)	ET
*Parahypoxylon papillatum*	ATCC 58729		USA	KC968919	KY610454	KY624223	KC977258	Kuhnert et al. (2014a; ITS, *TUB2*), Wendt et al. (2018; LSU, *RPB2*)	T
*P. ruwenzoriense*	MUCL51392		D. R. Congo	ON792786	ON954156	OP251039	ON813078	Cedeño-Sanchez et al. (2023)	T
*Pyrenopolyporus bambusicola*	BCC89355		Thailand	OP304856	OP304876	OP981624	OQ101839	Wongkanoun et. al. (2023)	HT
*Py cinereopigmentosus*	BCC89382		Thailand	OP304860	OP304882	OP981627	OQ101843	Wongkanoun et. al. (2023)	HT
*Py. hunteri*	MUCL 52673		Ivory Coast	KY610421	KY610472	KY624309	KU159530	Kuhnert et al. (2017a; *TUB2*), Wendt et al. (2018; ITS, LSU, *RPB2*)	ET
*Py. laminosus*	MUCL 53305		Martinique	KC968934	KY610485	KY624303	KC977292	Kuhnert et al. (2014b; ITS, *TUB2*), Wendt et al. (2018; LSU, *RPB2*)	HT
*Py. macrosporus*	BCC89373		Thailand	OP304870	OP304879	OP981621	OQ101844	Wongkanoun et al. (2023)	HT
*Py. nicaraguensis*	CBS 117739		Burkina Faso	AM749922	KY610489	KY624307	KC977272	Bitzer et al. (2008; ITS), Kuhnert et al. (2014a; *TUB 2*), Wendt et al. (2018; LSU, *RPB2*)	HT
*Py. papillatus*	BCC20324		Thailand HT	OP304854	OP304874	OP981619	OQ101846	Wongkanoun et al. (2023)	HT
*Py. tonngachangensis*	BCC31553		Thailand HT	OP304865	OP304887	OP981632	OQ101847	Wongkanoun et al. (2023)	HT
*Xylaria arbuscula*	CBS 126415		Germany	KY610394	KY610463	KY624287	KX271257	Fournier et al. (2011; ITS), Wendt et al. (2018; *TUB2*, LSU, *RPB2*)	
*X. hypoxylon*	CBS12260		Sweden	KY610407	KY610495	KY624231	KX271279	Sir et al. (2016; *TUB2*), Wendt et al. (2018; ITS, LSU, *RPB2*)	HT

*New taxa proposed in this study are in bold. ET, HT, and PT indicate epitype, holotype, and paratype, respectively. N/A, Data not available. Acronyms of culture collections: BCC, BIOTEC Culture Collection; Pathum Thani, Thailand; CBS, Centraalbureau voor Schimmelcultures, CBS-KNAW Culture, Utrecht, The Netherlands; EBS, Fundación Miguel Lillo, San Miguel de Tucumán, Argentina; MFLUCC, Mae Fah Luang culture collection; MUCL, Laboratory of Mycology, which is part of the Earth and Life Institute (ELI), in particular, the Pole of Applied Microbiology (ELIM) of the Université catholique de Louvain (UCLouvain); NBTF, National Biobank of Thailand, Pathum Thani, Thailand; STMA, HZI culture collection, Helmholtz Centre for Infection Research, Braunschweig, German.

### 2.4. Phylogenetic analyses

All sequences were aligned in the Multiple Sequence Comparison by Log-Expectation program (MUSCLE) (Edgar 2004) and manually refined. Multiple sequence alignments were analysed with closely matched sequences and other reference taxa obtained from GenBank and following the dataset of Cedeño-Sanchez et al. (2023) and Wongkanoun et al. (2023) as shown in Table 1. Sequences were analysed using maximum parsimony (MP), maximum likelihood (ML), and Bayesian algorithm (MB). The MP analysis was performed in PAUP * 4.0b10 (Swofford 2002), and all characters were equally weighted and gaps were treated as missing data. The most parsimonious trees were obtained from heuristic searches: 500 replicates of stepwise random addition and tree-bisection-reconnection (TBR) as a branch-swapping algorithm. Maximum parsimony bootstrap supports (BS) were estimated by 1,000 replicates (stepwise addition of sequence, 10 replicates of random addition of taxa, TBR branching-swapping algorithm). Tree length (TL), consistency index (CI), retention index (RI), relative consistency index (RC), and homoplasy index (HI) were estimated. The ML tree and bootstrap analyses were conducted through the CIPRES Science Gateway V. 3.3 (Miller et al. 2010) using Randomized Axelerated Maximum Likelihood (RAxML) 8.2.4 (Stamatakis 2014) with the BFGS method to optimise GTR rate parameters. Bayesian posterior probabilities of the branches were performed using MrBayes 3.0B4 (Huelsenbeck and Ronquist 2001) with the best-fit model (GTR + I + G) selected by AIC in Mr Modeltest 2.2 (Nylander 2004), tested with hierarchical likelihood ratios (hLRTs). Three million generations were run in four Markov chains and sampled every 100 generations with a burn-in value set at 3,000 sampled trees. Sequences of *Graphostoma platystomum* CBS 270.87 and *Xylaria arbuscula* CBS 126415 as well as *Xylaria hypoxylon* CBS 12260 were used as outgroups.

## 3. Results

### 3.1. Taxonomy

**Figure 1.**
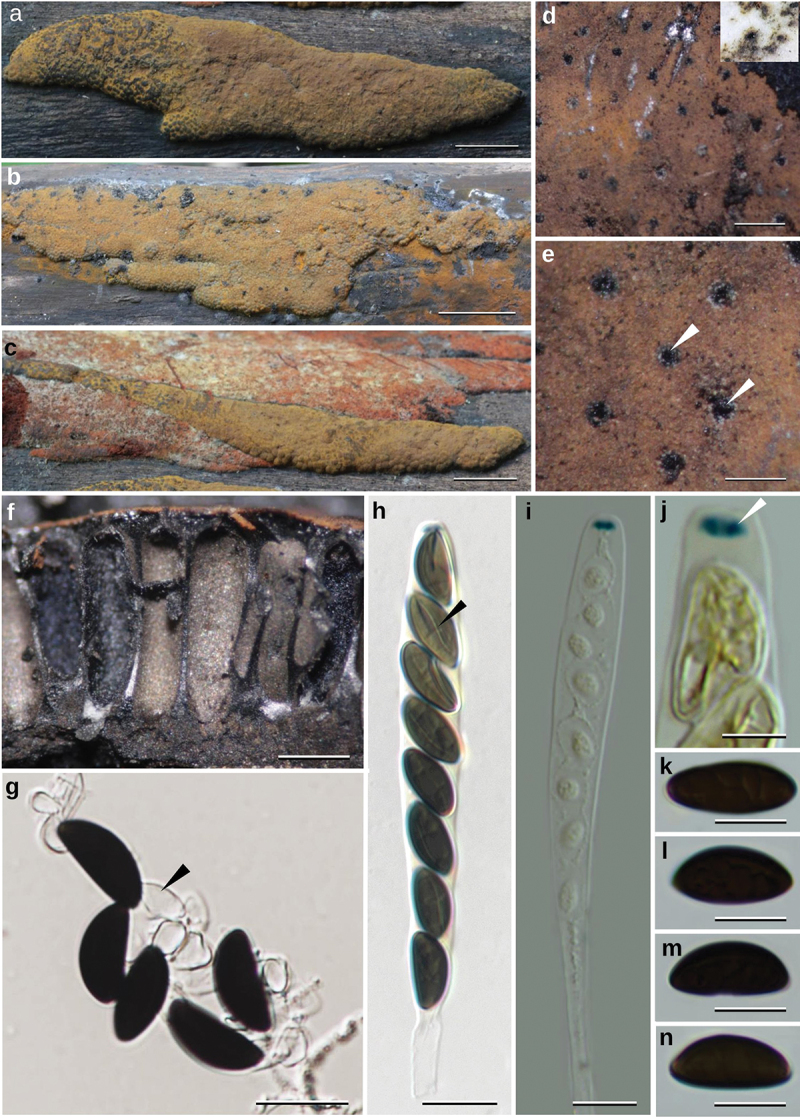
*Hypoxylon luteogranulatum* (BBH 40800). (a–b) Stromata in the natural habitat on wood. (c) Stromata associated with *Hypoxylon haematostroma*. (d) Stromatal surface with stromatal KOH-extractable pigments. (e) Stromatal surface shows ostioles (white arrows). (f) Longitudinal section of the stroma showing perithecia. (g) Ascospores in 10% KOH solution showing dehiscent perispore. (h) Ascus in Melzer’s reagent showing germ slit (black arrow). (i) Young ascus in Melzer’s reagent. (j) Apical apparatus bluing in Melzer’s reagent (white arrow). (k–n) Ascospores. Scale is indicated by bars: a, c = 10 µm; b = 20 mm; d = 400 µm; e = 200 µm; f = 0.5 mm; g–h = 20 µm; j = 5 µm; i, k–n = 10 µm.

**Figure 2.**
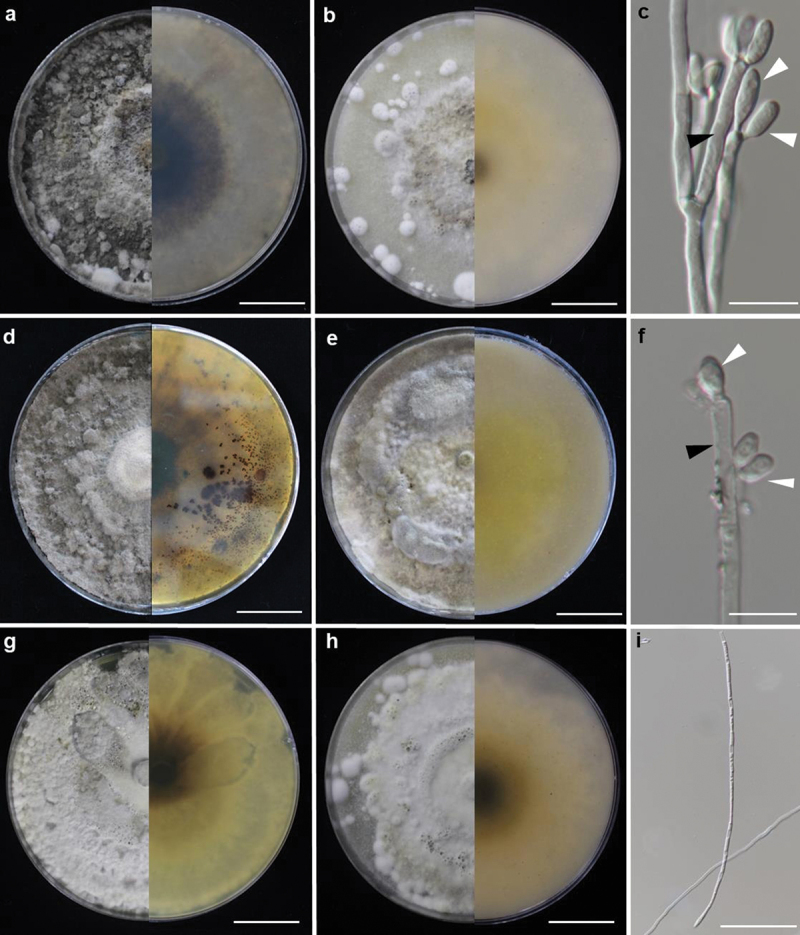
Culture characteristics after 2-week incubation of *Hypoxylon luteogranulatum*. *Hypoxylon luteogranulatum* BCC 91230: (a) culture on PDA, (b) culture on OA, (c) conidiogenous cells with Nodulisporium-like branching patterns, conidiogenous cell (black arrow), conidia (white arrows). *Hypoxylon luteogranulatum* BCC 79720: (d) culture on PDA, (e) culture on OA, (f) conidiogenous cells with Nodulisporium-like branching patterns, conidiogenous cell (black arrow), conidia (white arrows). *Hypoxylon luteogranulatum* BCC 91232: (g) culture on PDA, (h) culture on OA, (i) sterile hyphae. Scale is indicated by bars: a–b, d–e, g–h = 2 cm; c = 25 µm; f = 20 µm; i = 50 µm.

***Hypoxylon*** ***luteogranulatum*** Srikitikulchai, Wongkanoun, Charria-Girón, M. Stadler & Luangsa-ard sp. nov. Figures 1,2

MycoBank: MB 854508.

Etymology: *“luteogranulatum”* (Latin) refers to the yellow granules beneath the stromatal surface, combined with the observed yellow pigments in KOH.

Diagnosis: Differs from *Daldinia* species by having effused, pulvinate or peltate stromata. Differs from the species in *Pyrenopolyporus* by the lack of the massive tissue below the perithecial layer and by the absence of hypoxylone-like naphthoquinones. Differs from *Hypoxylon lechatii* by having a yellow stromatal surface and by having larger ascospores.

Type: Thailand: Chiang Mai Province, Huai Baba community forest, 19.070 ″N, 98.296 ″E, hill evergreen forest, associated with *Hypoxylon haematostroma*, on decaying wood, 22 November 2015, S. Wongkanoun, P. Srikitikulchai, (BBH 40800), (ex-BCC 79720); DNA sequences of ex-holotype strain ITS – PP955304, LSU – PP955701, *RPB2* – PP968827, *TUB2* – PQ000351.

Description: Teleomorph. *Stromata* widely effused-pulvinate or peltate, the base broadly attached to the substrate, conspicuous or inconspicuous perithecial mounds, 25–29 mm long, 9.45–13 (−27) mm broad, 2–3 mm thick; surface Grey Olivaceous (107) and Greenish Olivaceous (90) dark granules forming a thin crust above perithecia, with 10% KOH producing Vinaceous (57) extractable pigments, the tissue between perithecia grey or blackish brown, the tissue below perithecia layer grey, 1.2–2.4 mm thick. *Perithecia* monostichous, lanceolate, 0.14–0.28 mm broad, 1.40–1.42 mm high; ostioles black, umbilicate. *Asci* cylindrical, eight-spored, 178–201 µm in length, spore-bearing parts 86–93 µm long, 11–14 µm broad; with apical apparatus bluing in Melzer’s reagent, discoid, 3–4 × 1–2 µm. *Ascospores* dark brown to blackish brown, unicellular, ellipsoid, inequilateral with narrowly rounded ends, 16–17 (−21) × 7–8 (−9) µm (Me = 16.86 × 7.43 µm, *N* = 25) with straight to slightly oblique germ slit much less than spore length on convex size, perispore dehiscent in 10% KOH, smooth.

Culture characteristics: Colonies on OA reaching the edge of a 9 cm Petri dish in 1 week, at first whitish becoming velvety to felty, azonate with entire margin, Olivaceous (48) after 2 weeks incubation. Colonies on PDA covering Petri dish in 2 weeks at first whitish becoming Dark Herbage Green (69) and Dull Green (70) velvety to felty, azonate with entire margin. Anamorph on OA. Virgariella-like to (much more frequently) Nodulisporium-like branching patterns as defined in Ju and Rogers (1996). *Conidiophores* main axis hyaline and cell walls rough or smooth dark brown to blackish brown. *Conidiogenous* cells cylindrical, hyaline, finely roughened, 18–20 × 3–4 µm. *Conidia* hyaline, smooth, ellipsoid 5 − 8 × 3 − 4 µm (Me = 6.86 × 3.00 µm, *N* = 10).

Secondary metabolites: Stromata contain hypoxyvermelhotin A and BNT as main compounds.

Other material examined: Thailand: Chiang Mai Province, Ban Saluang Nok community forest, 19°012 ″N, 98°886 ″E, hill evergreen forest on decaying wood, 22 September 2019, P.S. (BCC91230, XY03806; BCC91232, XY03808).

Notes: *Hypoxylon luteogranulatum* strongly resembles *H. lechatii*, *H. anthochroum*, and *H. macrocarpum* in possessing brown vinaceous effused-pulvinate stromata and similar ascospores characters. However, it differs from the latter in having yellow pigments beneath the stromatal surface and much larger ascospores (Table 2) and lacks the papillate ostioles of *H. luteogranulatum*. Our newly described species can be distinguished from *H. lechatii* by the presence of a grey-olivaceous pigment on the stromatal surface in the KOH reaction and by having the largest ascospores: 16–17 (−21) × 7–8 (−9) µm for *H. luteogranulatum* vs. 8.5–10 × 4–4.5 µm for *H. lechatii. Hypoxylon anthochroum* can also be characterised in contrast to *H. luteogranulatum* by producing an isabelline pigment in KOH reaction and by having smaller ascospores: 8.5–13.5 × 4–6 µm vs. 16–17 (−21) × 7–8 (−9) µm for *H. luteogranulatum*. *Hypoxylon macrocarpum* differs from the Thai species by producing an isabelline to greyish-brown pigment in the stromatal KOH reaction and showing dark reddish-brown granules immediately beneath the stromatal surface. It also has smaller ascospores than *H. luteogranulatum*: 9–12.5 (−13) × 4–5.5 µm for *H. macrocarpum* vs. 16–17 (−21) × 7–8 (−9) µm for *H. luteogranulatum*.

**Table 2.** Comparison of morphological and chemotaxonomic characters of Hypoxylaceae species with massive stroma and long tubular perithecia and *Hypoxylon* species that are similar to *H. luteogranulatum.*

Taxon	Ascospore perispore	Ascospore germ slit	Ascospore size (μm)	KOH-extractable pigments	Metabolite (stroma)
*Daldinia chiangdaoensis*	Dehiscent	Straight germ slit spore-length on convex side	(13–) 15–18 (19) × (5–) 6–8 (−10)	Vinaceous grey	BNT, cytochalasins
*Daldinia korfii*	Dehiscent	Straight germ slit spore-length on convex side	(10.3–)11 − 14(−16.0) × (4.8–) 5.2 − 6.2 (−7)	Brown vinaceous to dark vinaceous	BNT, concentricol B and cytochalasin
*Daldinia phadaengensis*	Dehiscent	Straight germ slit spore-length on convex side	(11–) 14–16 (−18) × 5–6	Isabelline	BNT, daldinins A1 and A4
*Daldinia placentiformis*	Dehiscent	Spore length, dorsal	14.5–16 × 6.5–7	Olivaceous	BNT, napththols, naphthoquinones
*Daldinia kretzschmarioides*	Dehiscent	Spore length, dorsal	(12–) 13–16 × 5–6	Dilute purple or absent	BNT, cytochalasins
*Hypoxylon anthochroum*	Dehiscent	Straight to slightly sigmoid germ slit spore-length	8.5–13.5 × 4–6	Isabelline	N/A
*Hypoxylon begae*	Dehiscent	Short, dorsal	21–29 × 12–14.5	Dense isabelline	BNT, napththols, and unknown metabolite
*Hypoxylon lechatii*	Dehiscent	Straight germ slit spore-length	8.5–10 × 4–4.5	Brown vinaceous	BNT, hypoxyvermelhotin A
Hypoxylon luteogranulatum*	**Dehiscent**	**Straight to slightly curved germ slit spore-length on convex side**	**16–17 (−21) × 7–8 (−9)**	**Grey olivaceous**	**BNT, hypoxyvermelhotin A**
*Hypoxylon macrocarpum*	Dehiscent	Straight germ slit spore-length	9–12.5 (−13) × 4–5.5	Brown vinaceous	BNT, macrocarpones, and orsellinic acid
*Pyrenopolyporus laminosus*	Indehiscent	Spore length, dorsal	11–13.5 × 4.2–4.5	Dilute purple	BNT, napththols, naphthoquinones
*Pyrenopolyporus nicaraguensis*	Indehiscent	Spore length, dorsal	(11–) 12–15(−16) × 5–6.5	Dense purple or absent	BNT, napththols, naphthoquinones

*This study.

**Figure 3.**
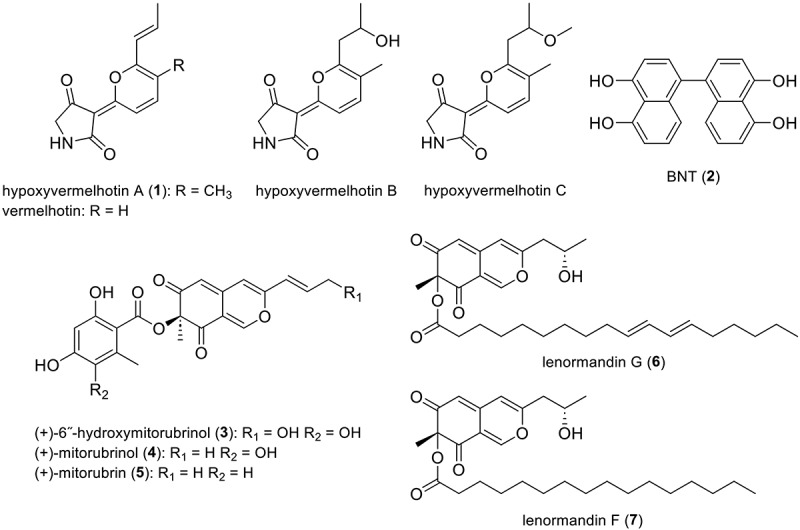
Chemical structures of stromatal metabolites detected in *Hypoxylon luteogranulatum* and related derivatives from *H. lechatii* (No numbered), as well as those detected on the stromata of *H. haematostroma*.

**Figure 4.**
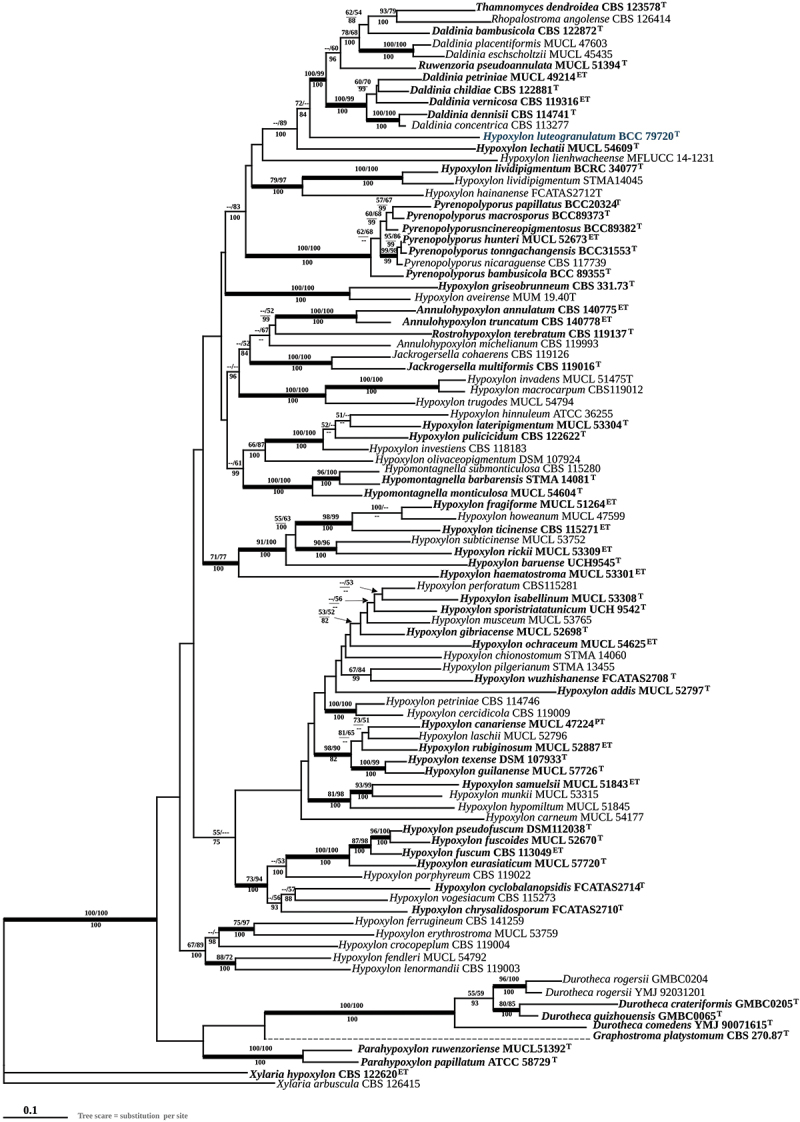
Phylogenetic relationships inferred from RAxML on *TUB2* sequence data of *Hypoxylon luteogranulatum* and other selected Xylariales. Support values from MP, ML, and Bayesian (MB) analyses higher than 50% (MP, ML) and 70% (MB) and are given above (MP/ML) and below (MB) the respective branches. Branches of significant support (BS 70% and PP 95%) are thickened. ET (ex-epitype), HT (ex-holotype), and PT (ex-paratype) strains are highlighted in bold letters.

**Figure 5.**
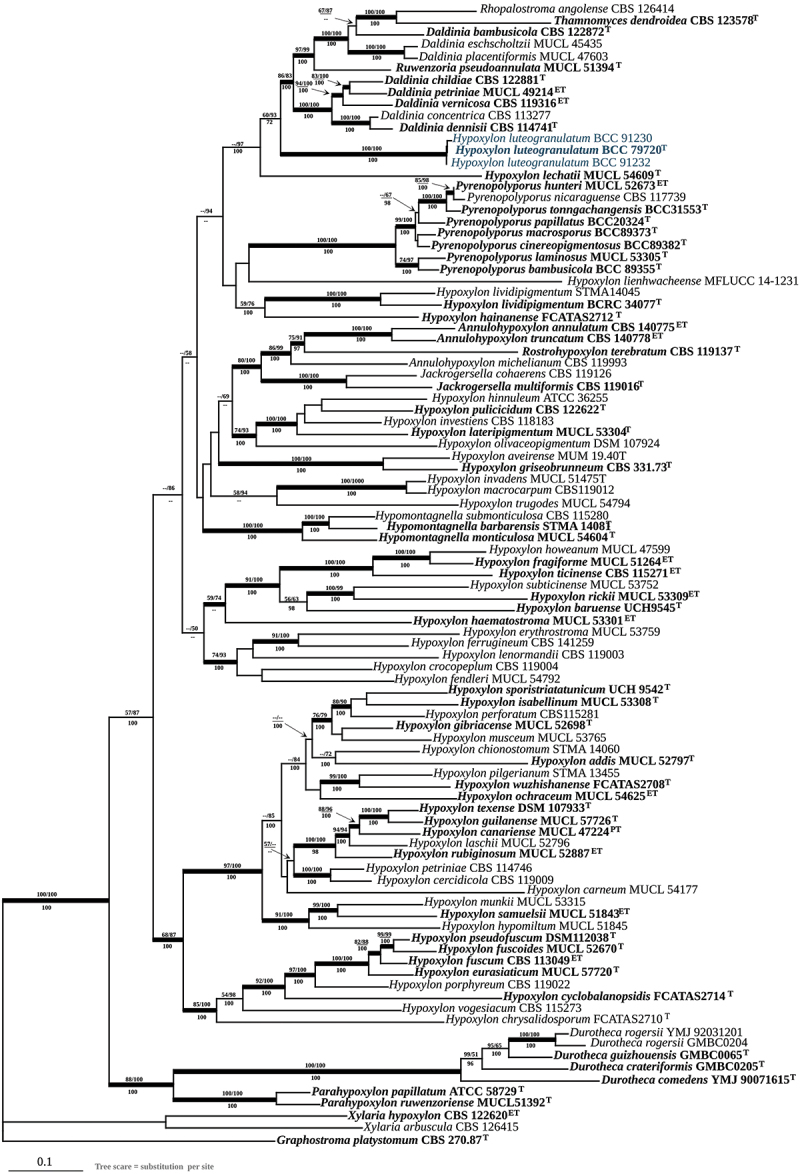
Phylogenetic relationships inferred from RAxML on the multi-locus alignment of *Hypoxylon luteogranulatum* and other selected Xylariales based on concatenated ribosomal (ITS and LSU) and proteinogenic (*TUB2* and *RPB2*) DNA sequence data. Support values from MP, ML, and Bayesian (MB) analyses higher than 50% (MP, ML) and 70% (MB) are shown above (MP/ML) and below (MB) the respective branches. Branches of significant support (BS 70% and PP 95%) are thickened. ET (ex-epitype), HT (ex-holotype), and PT (ex-paratype) strains are highlighted in bold letters.

The occurrence of vermelhotin-type tetramic acids is very unique in *Hypoxylon* (Kuhnert et al. 2014). The new species can easily be segregated from *H. macrocarpum* by the lack of macrocarpones (Figure 3) as well as from all chemotypes of *H. anthochroum* sensu Ju and Rogers (1996), by the lack of daldinins (Figure 3). It can also be easily distinguished from *Pyrenopolyporus* species by the absence of hypoxylone-like naphthoquinones (Figure 3). As shown in the figure (Figure 5) the phylogenetic position of our new fungus is very stable with high statistical support of bootstrap analyses of maximum likelihood (BSML). Especially the beta-tubulin DNA sequence shows a closer relationship to *Daldinia* spp. than to other *Hypoxylon* spp. and we found it clearly segregated from *H. lechatii* (Figure 4). Furthermore, our findings based on multi-locus analyses confirmed this fungus as a new species of *Hypoxylon*.

### 3.2. Molecular phylogeny

After providing the full taxonomic description of the new fungus from Thailand, we confirmed its phylogenetic placement using multi-locus DNA analyses as shown in Figure 5 and single-gene DNA analyses as shown in Figures S1–S4. The 10 newly generated ITS, LSU, and *RPB2* sequences were compared with data from the GenBank NCBI nucleotide database (PCR amplifications yielded approximately 500 bp, 1,000 bp, 800 bp, and 1,000 bp of ITS rDNA, LSU rDNA, *RPB2*, *TUB2* sequences, respectively). The phylogenetic relationships were estimated using the MP, ML, and MB analyses. The dataset of the multi-locus DNA sequences included 93 taxa from the Hypoxylaceae: *Annulohypoxylon* (3), *Daldinia* (8), *Durotheca* (5), *Hypomontagnella* (3), *Hypoxylon* (59), *Jackrogersella* (2), *Parahypoxylon* (2), *Pyrenopolyporus* (8), *Rhopalostroma* (1), *Ruwenzoria* (1), and *Thamnomyces* (1). The combined dataset consisted of 6,431 characters, of which 2,974 were constant, 2,356 were parsimony informative, and 1,099 were uninformative. The MP analysis yielded 3 trees with a CI of 0.258, a RI of 0.487, and a HI of 0.742. The best phylogenetic tree inferred from RAxML had a likelihood of −105318.985. The alignment had 2,456 distinct alignment patterns, with 23.49% undetermined characters or gaps. Estimated base frequencies were as follows: A = 0.239, C = 0.264, G = 0.261, T = 0.234; substitution rates were AC = 0.951, AG = 3.656, AT = 1.433, CG = 0.668, CT = 4.801, GT = 1.000; gamma distribution shape parameter was α 0.403. The likelihood of the Bayesian tree was −105438.970. As shown in Figure 4, the sequences of the new Thai strains are well separated from the previously reported the genera in Hypoxylaceae that have been recently updated by Wendt et al. (2018) and Cedeño-Sanchez et al. (2023). As the topology of the RAxML tree is practically identical to the one presented by Cedeño-Sanchez et al. (2023) from which most DNA sequence data were included in our study and analysed using essentially the same methodology, we restrict our discussion on the phylogenetic positions of the new taxa.

### 3.3. *Chemical and ecological traits of* Hypoxylon luteogranulatum

Stromata of three herbarium specimens of *Hypoxylon luteogranulatum* were extracted and analysed by UHPLC-DAD-IM-MS/MS. The obtained raw data were pre-processed, and the respective feature table was dereplicated by comparing the high-resolution *m/z*, MS/MS spectra, retention time, collision cross-section (CCS) value, and UV/Vis spectra with reference data from our in-house library of common stromatal metabolites of Hypoxylaceae species (Cedeño-Sanchez et al. 2023). However, aside from BNT, no other metabolites were annotated or yielded significant MS/MS similarity with compounds belonging to known compound classes from stromatic Hypoxylaceae in our library.

Among the other major metabolites, a compound eluting at 3.69 min (**1**, *m/z* = 232.0966 Da), with a molecular formula predicted as C_13_H_13_NO_3_, was tentatively identified as hypoxyvermelhotin A, a tetramic acid only found in the stromata of *H. lechatii* (Figure 3). To further validate this unique metabolite in the Hypoxylaceae, we targeted the isolation of this compound. Therefore, compound **1**, obtained as a yellow-to-orange powder, was identified as hypoxyvermelhotin A based on a comparison of its UV, MS, and NMR data (Figure S5–S6) to the original report by Kuhnert et al. (2014).

Interestingly, one of the collections from *H. luteogranulatum* was found to be associated with *H. haematostroma*. This represents a rare phenomenon in the Hypoxylaceae, with only a few such cases reported (Becker et al. 2020). There are some species of *Hypoxylon* associated with the old stromata of other fungi. *Hypoxylon lechatii* has been found on the bark of an unknown host, associated with the old stromata of *Camillea heterostoma*. Additionally, stromata of *H. aeruginosum* were found to colonise overmature stromata of *H. musceum*, and *H. samuelsii* was found to be associated with the old stromata of *Kretzschmaria* sp. on dead wood. This reinforces the suspicion of a fungicolous lifestyle as noted by Læssøe et al. (2010) and Fournier and Lechat (2015). However, with the current data, it is not possible to identify the type of interaction between *H. luteogranulatum* and *H. haematostroma* or if this phenomenon is a coincidence resulting due to overlapping host ranges.

Therefore, we decided to gain insights at the chemical level using state-of-the-art metabolomics. Raw data files from the stromatal extracts of each fungus and their interaction zone were imported into MetaboScape software and processed together, which resulted in 86 features at the MS level. Five further compounds only present in the extracts from *H. haematostroma* and the interaction zone were annotated as (+)-6˝-hydroxymitorubrinol (**3**), (+)-mitorubrinol (**4**), (+)-mitorubrin (**5**), lenormandin G (**6**), and lenormandin F (**7**). Remarkably, lenormandins had so far been only found in *H. lenormandii* and closely related taxa (Kuhnert et al. 2015). However, a comparison with authentic standards from our MS/MS spectral library of secondary metabolites from hypoxylaceous taxa confirmed their presence in *H. haematostroma*. To further verify the taxonomical identification of this fungus, we compared the stromatal extracts of the Thai specimen with the epitype of *H. haematostroma*, validating the presence of these metabolites in its stromata.

**Figure 6.**
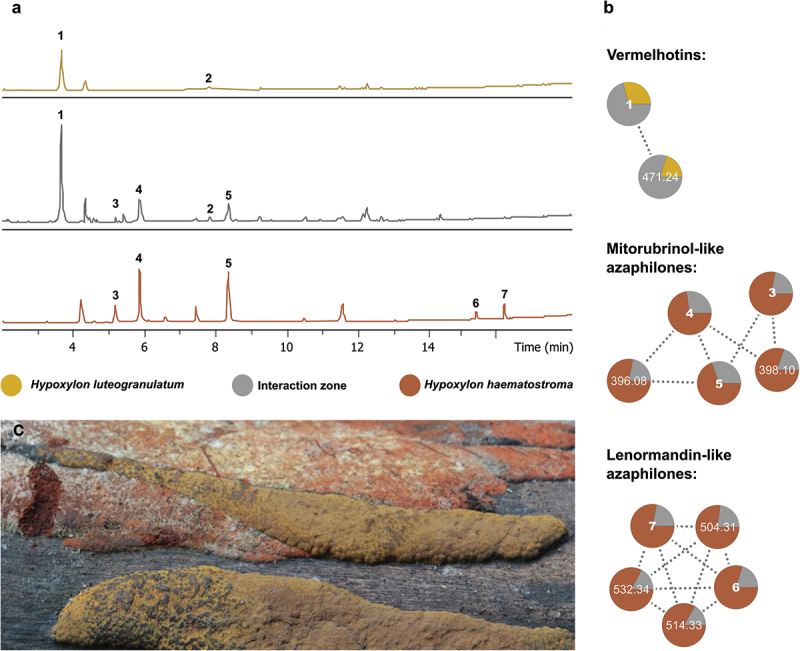
(a) Base peak chromatograms (BPCs) of stromatal extracts of *Hypoxylon luteogranulatum* and *H. haematostroma*, found to be associated in nature. Identified metabolites are depicted by bold numbers over their corresponding peaks: hypoxyvermelhotin a (**1**), BNT (**2**), (+)-6˝-hydroxymitorubrinol (**3**), (+)-mitorubrinol (**4**), (+)-mitorubrin (**5**), lenormandin G (**6**), and lenormandin F (**7**). (b) Molecular families related to vermelhotins, and mitorubrinol- and lenormandin-like azaphilones found in the stromata of *H. luteogranulatum* and *H. haematostroma*, as well as in their interaction zone. (c) Stromata of *H. luteogranulatum* and *H. haematostroma* in the natural habitat.

Afterwards, a Feature-Based Molecular Networking (FBMN; Figure 6) analysis was conducted to explore the chemical diversity of the identified compounds and their relative intensity in the different samples. From the 82 features detected at the MS/MS level, we identified 12 molecular families (MFs) with at least 2 connected nodes, along with 33 singletons. Notably, most of the induced features could be traced back to the stromata of *Hypoxylon luteogranulatum*. However, when restricting the analysis to the major metabolites, hypoxyvermelhotin A (**1**) is apparently induced in the contact zone between these two fungi, which might indicate a biological function in this interaction. In contrast, focusing on the two molecular families of identified azaphilones reveals that these compounds are also present in the interaction zone, but their relative intensity is higher in the stromata of *H. haematostroma*. While no other known vermelhotin-like molecules were found through our analysis, a feature eluting at 5.44 min (*m/z* = 472.2438 Da), with a molecular formula predicted as C_30_H_33_NO_4_, was found to be clustered together with hypoxyvermelhotin A. The MS/MS spectrum of this compound resembles that of compound **1**, generating main fragment ions at 232.0967 Da and 205.0858 Da, which represent the core structure of hypoxyvermelhotin A. The difference between these two molecules is indicated by a neutral loss interpreted as C_17_H_20_O. The absence of further significant fragments in the spectra prevents us from gaining additional structural information about the substitution occurring in this potential new vermelhotin derivative. It is noting that while the present results suggest a plausible biological role for the studied metabolites in the interaction between both species, further quantitative analysis is needed to establish a link between the production of vemelhotins and the putative fungicolous behaviour of *H. luteogranulatum*.

**Figure 7.**
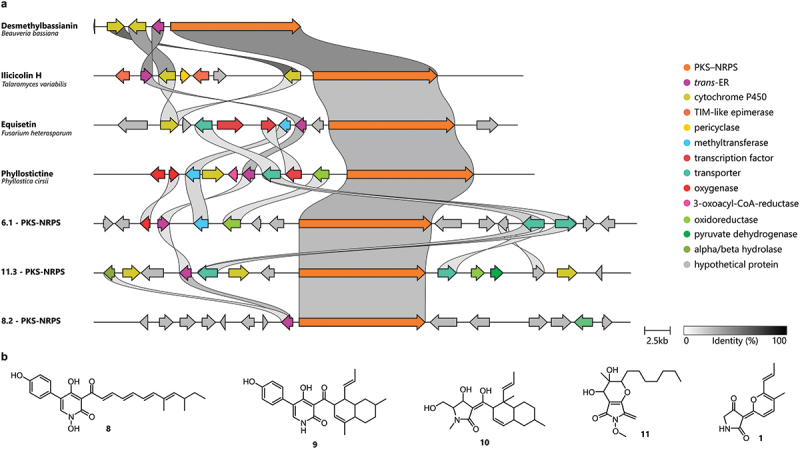
(a) Synteny analysis of the three PKS-NRPS hybrid cluster identified in *Hypoxylon lechatii* and gene clusters involved in the biosynthesis of desmethylbassianin (**8**; Altimira et al. 2022), ilicicolin H (**9**; Zhang et al. 2019), equisetin (**10**; Sims et al. 2005), and phyllostictine a (**11**; Trenti and Cox 2017). (b) Their corresponding chemical structures.

To gain insights into the biosynthetic origin of these molecules, we investigated the presence of these pathways in the genome of *H. lechatii*, which recently became available during ongoing phylogenomic studies (Cedeño-Sanchez et al. 2024a). Even though metabolites originating from PKS-NRPS are relatively rare within the Hypoxylaceae (Kuhnert et al. 2021), three out of the 57 biosynthetic gene clusters (BGCs) predicted by antiSMASH 7.1.0 were putative PKS-NRPS hybrid clusters. When comparing the identified clusters with related BGCs involved in the biosynthesis of tetramic acids/2-pyridones, we observed that they contained a varying number of oxidoreductases, tailoring genes, transporters, transcription factors, and regulatory genes besides the core PKS-NRPS hybrid genes (Figure 7). From these clusters, the cluster located at the contig 6 and region 1 (6.1) was found to share varying homology with the genes within the bicyclic 3-methylene tetramic acid phyllostictine A gene cluster. This BGC includes an *O*-methyltransferase-encoding gene, which would be required for *O*-methylation of vermelhotin to form hypoxyvermelhotin C and is not present in the other PKS-NRPS BGCs. This particular BGC encompasses 16 open reading frames encoding potential biosynthetic proteins. Intriguingly, the closest pBLAST hits correspond to genes found in genomic sequences belonging to species from the genus *Daldinia.*

Based on the biosynthetic relationship of vermelhotin-like molecules to other tetramic acids, a retrobiosynthesis pathway was proposed as shown in Figure S7. We hypothesised that hypoxyvermelhotin C, putatively the final pathway product, is obtained through an *O*-methylation catalysed by the *O*-methyltransferase, while hypoxyvermelhotin B is obtained via hydroxylation at the double bond on the side chain of **1**. The formation of the 3-(2 H-pyran-2-ylidene) pyrrolidine-2,4-dione skeleton might occur via oxidative cyclisation of the linear product of the PKS-NRPS and *trans-*enoyl reductase (ER), analogous to the biosynthesis of the *Fusarium* mycotoxin (-)-sambutoxin (Go et al. 2021). However, further efforts are required to elucidate the function of the individual genes within the identified BGC.

## 4. Discussion

Our multi-locus phylogenetic analyses show that *Hypoxylon luteogranulatum* formed a sister group with *Daldinia* spp., which based on morphological concept is characterised by the development of internal concentric zones below the perithecia outline (Ju and Rogers 1996; Stadler et al. 2014). The stromata of certain *Daldinia* spp., such as *D. placentiformis*, *D. korfii*, and *D. kretzschmarioides* share similar features with *Hypoxylon*, in lacking the internal concentric zones. Nevertheless, the similarities between these species and *Daldinia* were verified through the analysis of ITS and *TUB2* sequences, as well as the presence of cytochalasans and concentricol B in the stromata of *D. korfii* (Sir et al. 2016). These compounds serve well as chemotaxonomic markers for *D. concentrica*, *D. eschscholtzii*, and certain members of the *D. eschscholtzii* group (Quang et al. 2002; Stadler et al. 2014). *Daldinia kretzschmarioides* shares a strong morphological resemblance to certain species of *Hypoxylon*. However, integrative taxonomic studies confirmed its affinity to the genus *Daldinia* (Wongkanoun et al. 2019). Recently, some *Daldinia* species were reported that are only known from anamorphic traits, and nobody knows how their telemorphs look like (cf. Yin et al. 2024).

*Pyrenopolyporus* is clearly separated from our new fungus based on both multi-locus and beta-tubulin phylogenetic analyses. Morphologically, *Pyrenopolyporus* differs from our new species by having a massive tissue below the perithecial layer and presenting perispore indehiscent in KOH solution. The stromatal KOH-extractable pigments mainly show purple colouration, corresponding to the production of BNT and naphthoquinone derivatives, which are chemotaxonomic markers of species from this genus (Wongkanoun et al. 2023). *Hypoxylon lienhwacheense* is also closely related to *H. luteogranulatum* based on the beta-tubulin phylogenetic analysis. This rare species was first reported by Ju and Rogers (1996), with its type specimen collected from Taiwan of China as specimen no. WSP 69625. The morphological features of this fungus are clearly distinguished from *H. luteogranulatum* by having smaller ascospores and producing violet pigments in 10% KOH solution. Contrary to the beta-tubulin analysis, our multi-locus phylogeny shows that *H. lienhwacheense* is more closely related to *Pyrenopolyporus* spp., even though both present completely different stromatal secondary metabolites (Sir et al. 2016).

One of the most interesting features encountered in *H. luteogranulatum* is its stromatal metabolites, which are almost exclusively vermelhotin-type moleculees. In fact, tetramic acids and their related 2-pyridones are widely found in different lineages of the Ascomycota, however, vermelhotin derivatives were first reported from endophytic fungi classified within the order Pleosporales, a group distantly related to the Xylariales (Hosoe et al. 2006; Kasettrathat et al. 2008; Leyte-Lugo et al. 2012). Interestingly, vermelhotin-like molecules are the major metabolites found in the stromata of *H. lechatii*, and no other tetramic acids or related 2-pyridones have ever been detected in stromata of the Xylariales. The ecological function of vermelhotins and their involvement in the putative fungicolous lifestyle of *H. luteogranulatum* remains unclear, and further efforts must be dedicated to unveil the chemical and biological basis of this rare phenomenon.

*Hypoxylon lechatii*, characterised by effused pulvinate and brown stromata associated with old *Camillea heterostoma*, was first collected in French Guiana (Kuhnert et al. 2014). The major stromatal metabolites of this species are BNT and derivatives of the tetramic acid vermelhotin (Figure 3), previously known from fungal endophytes (Hosoe et al. 2006; Kasettrathat et al. 2008; Leyte-Lugo et al. 2012; Kuhnert et al. 2014). The phylogenetic position of *H. lechatii* remains unclear, as it forms a sister species with *Daldinia* although no morphological or chemical evidence supports this relationship (Kuhnert et al. 2014), which was confirmed by using phylogenomic analyses (Cedeño-Sanchez et al. 2024a). Studies by Kuhnert et al. (2014) and Cedeño-Sanchez et al. (2024a) indicate that beta-tubulin data provide a more accurate depiction of *Hypoxylon* taxonomy compared to ITS sequences. Our molecular analysis confirmed that our sample is closely related to *H. lechatii*, but the morphological features of our new fungus are clearly different from the latter, having larger ascospores and showing pale yellow stomatal colour (see in the taxonomic part).

## 5. Conclusions

The present study focused on the taxonomy of hypoxylaceous taxa in Thailand and the description of a new species within this family, using a polyphasic approach that integrates molecular, morphological, and chemotaxonomic data. Comparative studies between *H. luteogranulatum* and *H. lechatii* at the genomic and metabolomic levels are needed to depict the intricate differences and/or similarities of these taxa. Despite significant advancements in modern technologies and their widespread application in taxonomy, we adhere to the original taxonomy based on the one fungus one name (1F1N) nomenclature system. Indeed, we advocate for the importance of the isolation of pure cultures for studies across different research fields, particularly regarding their biotechnological applications. We expect that the integration of multi-omics approaches will enable us to fully explore the potential of these.

## Supplementary Material

Hypoxylon luteogranulatumSI.pdf

## Data Availability

All sequence data generated for this work can be accessed via GenBank: https://www.ncbi.nlm.nih.gov/genbank/.
