# Supplementary material for: Hypoxylon luteogranulatum (Hypoxylaceae, Xylariales), a novel species from Thailand with distinct chemical and ecological traits
Source: Mycology. 2025 Jan 16;16(3):1296–314. doi: 10.1080/21501203.2024.2435979 (PMC12422043; doi:10.1080/21501203.2024.2435979)
Supplement: Hypoxylon luteogranulatumSI.pdf [file TMYC_A_2435979_SM5248.pdf]

Hypoxylon luteogranulatum (_Hypoxylaceae, Xylariales_), a novel species from Thailand with distinct chemical and ecological traits

Sarunyou Wongkanoun[1,2], Esteban Charria-Girón[4,5*], Marjorie Cedeño[4,5], Boonchuai Chainuwong[2], Sayanh Somrithipol[3], Eric Kuhnert[6], Prasert Srikitikulchai[2], Natapol Pornputtapong[1], Frank Surup[4,5], Jennifer Luangsa-ard[3], Marc Stadler [4,5]*

[1]Department of Biochemistry and Microbiology, Center of Excellence for DNA Barcoding of Thai Medicinal Plants, Faculty of Pharmaceutical Sciences, Chulalongkorn University, Bangkok 10330, Thailand

[2]National Biobank of Thailand (NBT), National Center for Genetic Engineering and Biotechnology (BIOTEC), 111 Thailand Science Park, Phahonyothin Road, Khlong Nueng, Khlong Luang, Pathum Thani 12120, Thailand

[3]Plant Microbe Interaction Research Team (APMT), Integrative Crop Biotechnology and Management Research Group, National Center for Genetic Engineering and Biotechnology (BIOTEC), 113 Thailand Science Park, Phahonyothin Road, Khlong Nueng, Khlong Luang, Pathum Thani 12120, Thailand

[4]Department of Microbial Drugs, Helmholtz Centre for Infection Research GmbH (HZI), Inhoffenstraße 7, 38124 Braunschweig, Germany;) German Centre for Infection Research Association (DZIF), partner site Hannover-Braunschweig, Inhoffenstraße 7, 38124 Braunschweig, Germany

[5]Institute of Microbiology, Technische Universität Braunschweig, Spielmannstraße 7, 38106 Braunschweig, Germany

[6]Ginkgo Bioworks, Inc., 27 Drydock Avenue, Boston, MA 02210, USA

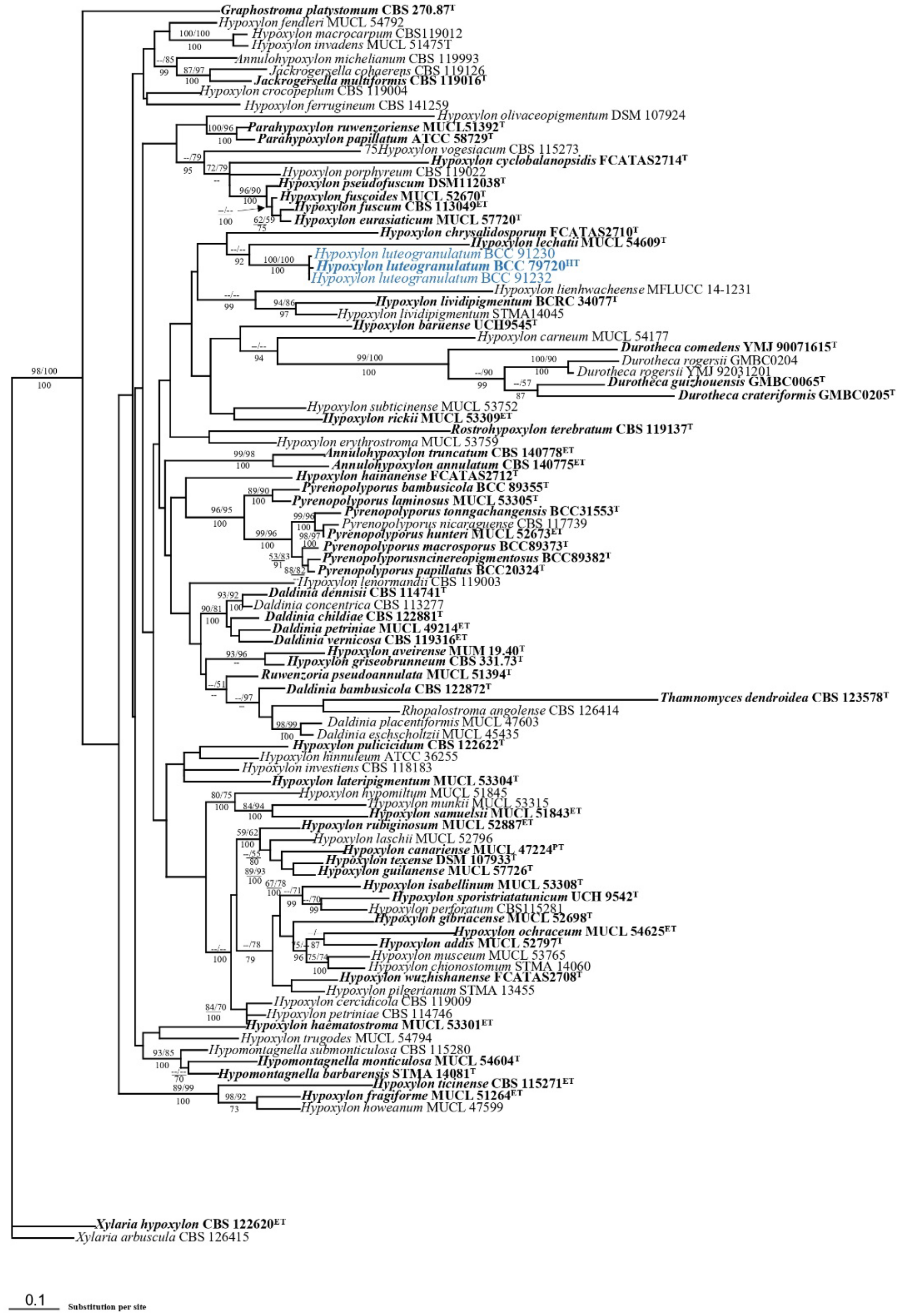

Fig. S1 Phylogenetic relationships inferred from RAxML on the internal transcribed spacer regions alignment of *Hypoxylon luteogranulatum* and other selected *Xylariales* based on ribosomal ITS DNA sequence data. Support values from MP, ML and Bayesian (MB) analyses higher than 50% (MP, ML) and 70% (MB) are shown above (MP/ML) and below (MB) the

respective branches. The black arrow indicates the sequences of *H. luteogranulatum* (in blue front). ET (ex-epitype), HT (ex-holotype), and PT (ex-paratype) strains are highlighted in bold letters.

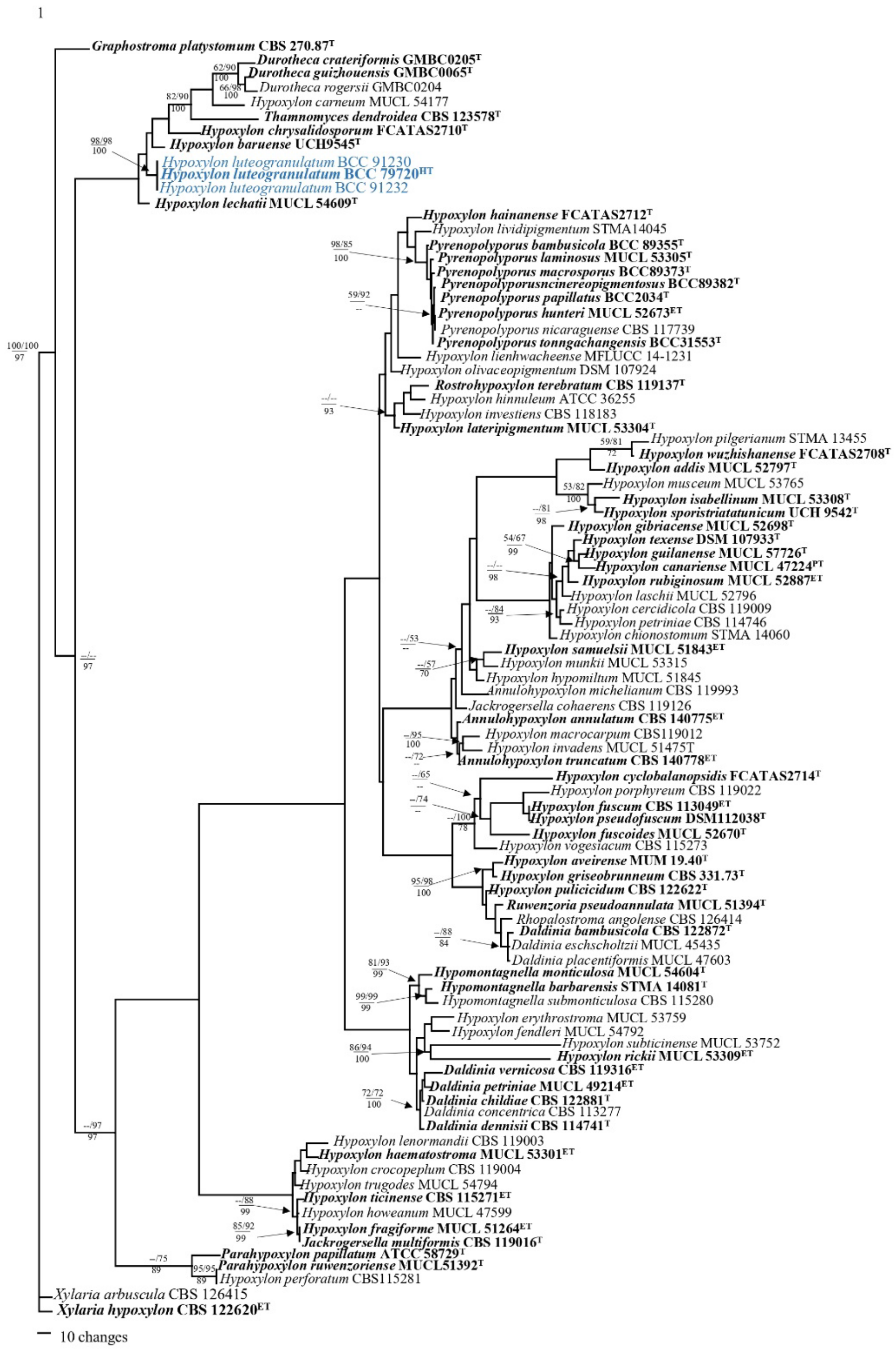

Fig. S2 Phylogenetic relationships inferred from Maximum parsimony tree on large subunit of the rDNA (LSU) alignment of *Hypoxylon luteogranulatum* and other selected *Xylariales* based on nuclear ribosomal LSU sequences data. Support values from MP, ML and Bayesian (MB) analyses higher than 50% (MP, ML) and 70% (MB) are shown above (MP/ML) and below (MB) the respective branches. The black arrow indicates the sequences of *H. luteogranulatum* (in blue front). ET (ex-epitype), HT (ex-holotype), and PT (ex-paratype) strains are highlighted in bold letters.

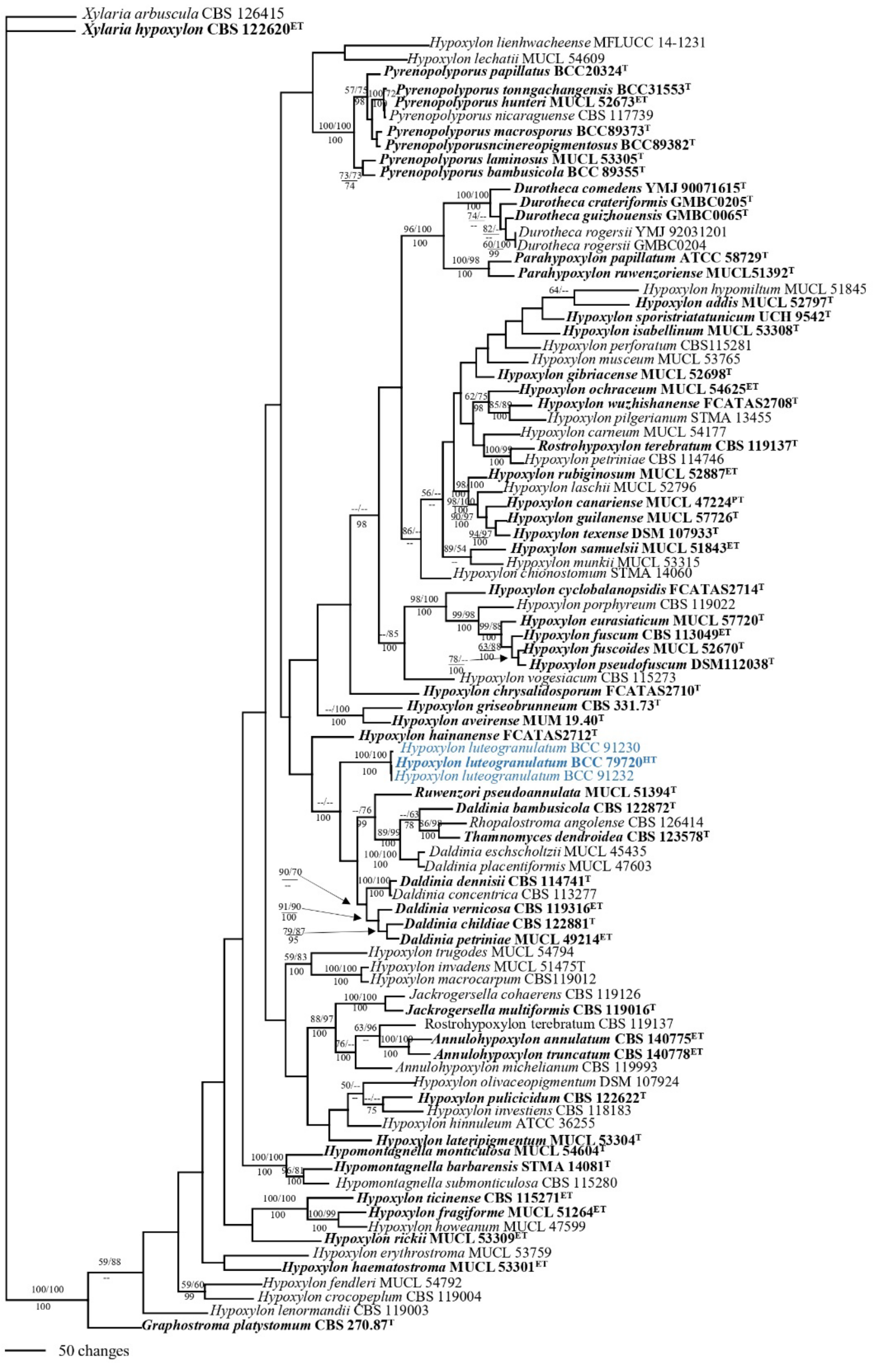

Fig. S3 Phylogenetic relationships inferred from Maximum parsimony tree on RNA polymerase II (*RPB*2) alignment of *Hypoxylon luteogranulatum* and other selected *Xylariales* based on proteinogenic *RPB*2 sequences. Support values from MP, ML and Bayesian (MB) analyses higher than 50% (MP, ML) and 70% (MB) are shown above (MP/ML) and below (MB) the respective branches. The black arrow indicates the sequences of *H. luteogranulatum*

(in blue front). ET (ex-epitype), HT (ex-holotype), and PT (ex-paratype) strains are highlighted in bold letters.

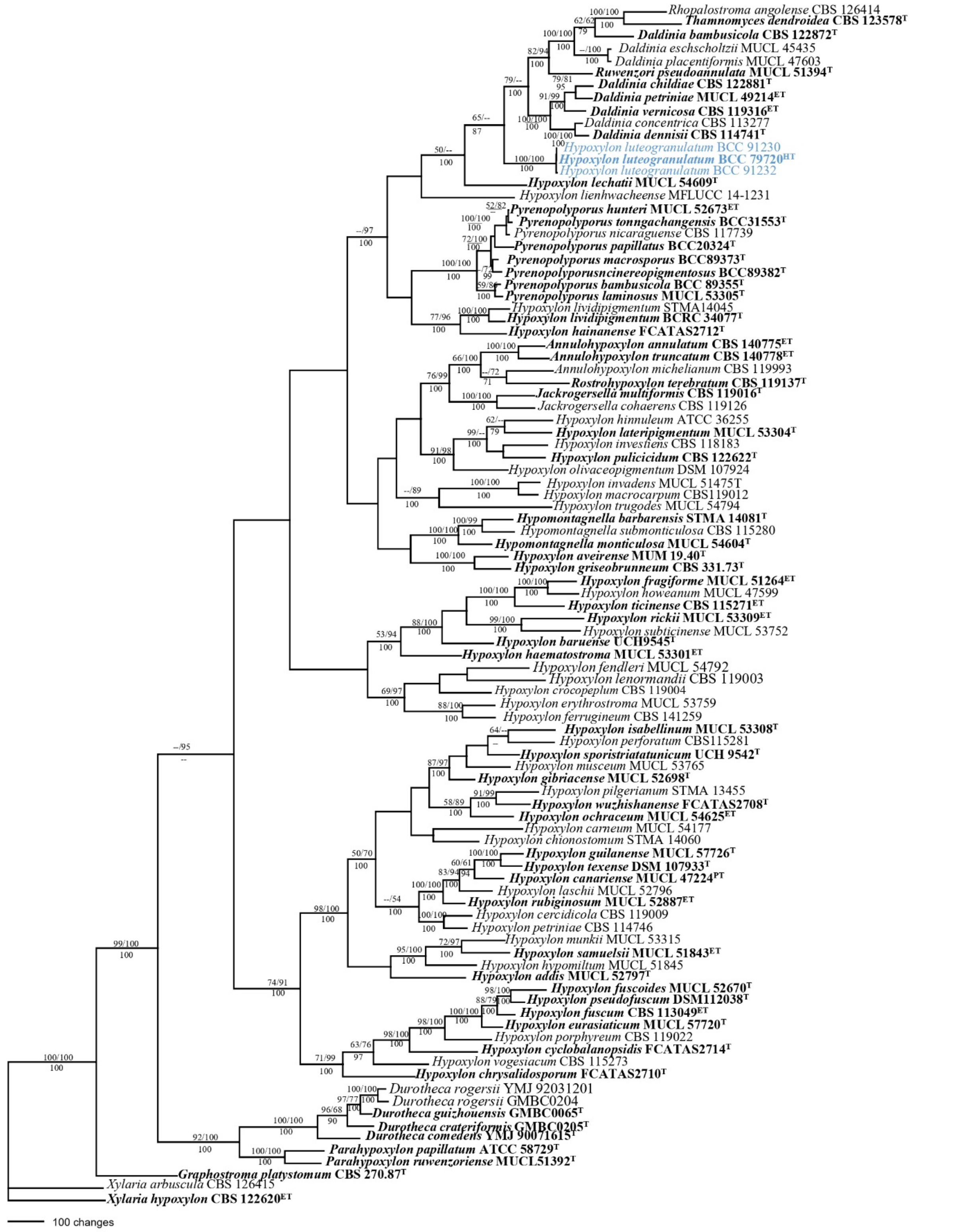

Fig. S4 Phylogenetic relationships inferred from RAxML on multi-locus alignment of *Hypoxylon luteogranulatum* and other selected *Xylariales* based on concatenated large subunit of the rDNA (LSU) and proteinogenic (*TUB2* and *RPB2*) DNA sequence data. Support values from MP, ML and Bayesian (MB) analyses higher than 50% (MP, ML) and 70% (MB) are shown above (MP/ML) and below (MB) the respective branches. The black arrow indicates the sequences of *H. luteogranulatum* (in blue front). ET (ex-epitype), HT (ex-holotype), and PT (ex-paratype) strains are highlighted in bold letters.

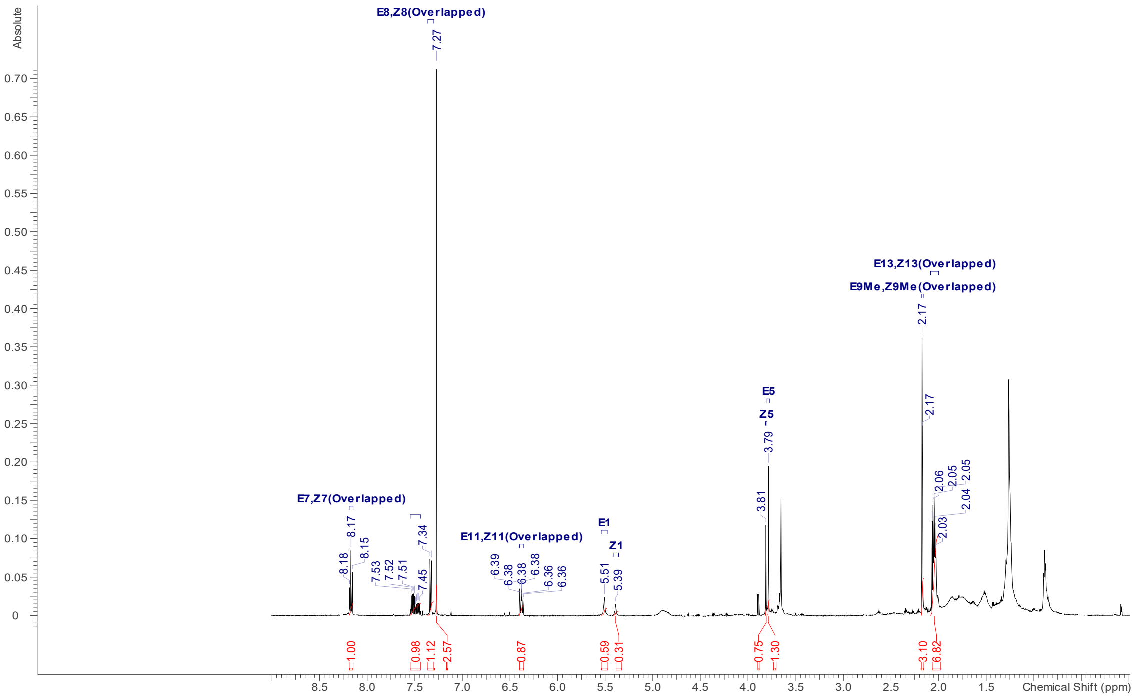

Fig. S5 ^{1}H NMR spectrum (700 MHz) of hypoxyvermelhotin in $CHCl_3$-*d*.

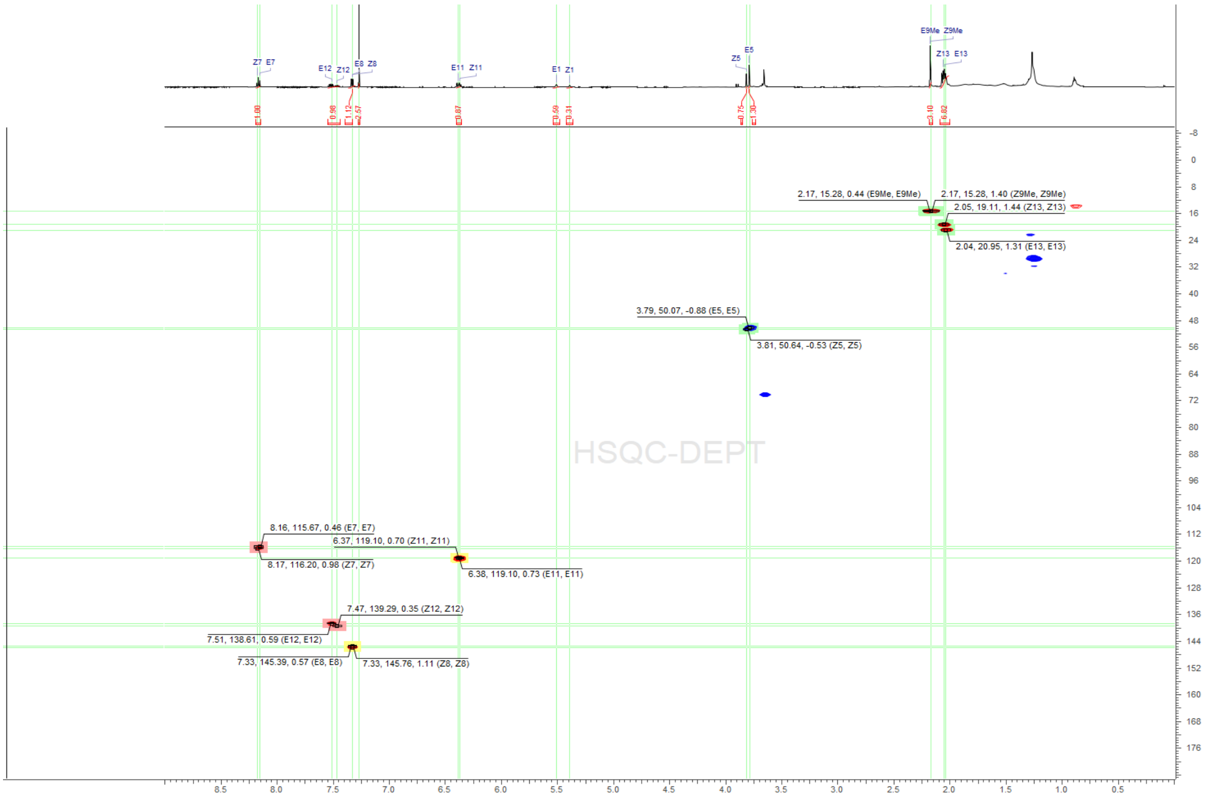

Fig. S6 HSQC NMR spectrum (700 MHz) of hypoxyvermelhotin in $CHCl_3$-d.

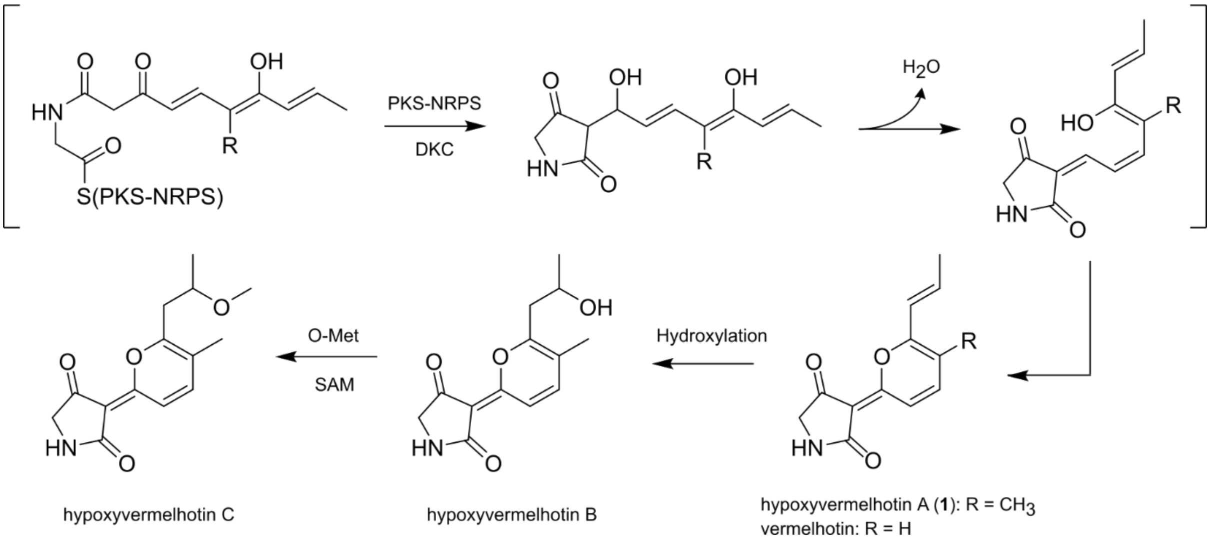

Fig. S7 Proposed biosynthesis of vermelhotin and hypoxyvermelhotin derivatives produced by *H. lechatii* and *H. luteogranulatum.* In brackets are shown proposed intermediates of the pathway.
